# Challenges of Joint Crisis Plans with Migrant Patients: Focus Groups with Mental Health Professionals and Interpreters

**DOI:** 10.1007/s10597-025-01523-3

**Published:** 2025-11-04

**Authors:** Orest Weber, Imane Semlali, Vincent Wenger, Pascale Ferrari, Sergio Felix Mota, Susana Ramos Almeida, Alexandra Brodard, Felicia Dutray

**Affiliations:** 1 Community Psychiatry Service, Department of Psychiatry, Lausanne University Hospital, Pl. Chauderon 18, 1003 Lausanne, Switzerland; 2 Rue Des Terreaux 10, 1003 Lausanne, Switzerland; 3 Vetroz, Switzerland; 4 La Source School of Nursing, HES-SO University of Applied Sciences and Arts Western Switzerland, Av. Vinet 30, Lausanne, Switzerland

**Keywords:** Joint crisis plan, Shared decision making, Migrant, Interpreter, Mental health

## Abstract

In mental health, joint crisis plans facilitate advance care planning between clinicians and patients who experience psychiatric crises. Little research focuses on advance care planning with migrants with mental health problems. This study investigated clinician and interpreter perspectives on the use of joint crisis plans in intercultural and multilingual settings. Focus groups were conducted: two with mental health clinicians (6 participants per group) and two with interpreters (5 to 6 participants per group). The participants in the study identified several challenges associated with using joint crisis plans for patients with a migration background: Diverging ideas about mental health etiologies and roles in decision-making processes; patients’ fears of undesirable interferences between joint crisis plans and administrative procedures for residence permits; language barriers; and the collaboration with interpreters. However, interpreters are also found to play a central role by fostering patients’ trust in the clinicians and the joint crisis plan.

## Introduction

International health policies are increasingly moving towards a patient-centered care model, particularly through patients’ involvement in their own care planning (Linder, 2018; Pachoud, 2018; Vahdat et al., 2014). Meaningful participation of patients in decisions about their treatment appears to have a strong positive impact on their recovery (Covvey et al., 2019; Vahdat et al., 2014).

In the mental health sector, many patients experience crises requiring sensitive decisions on hospitalization, injection of medicine, the use of intensive care rooms, etc. Due to their acute psychiatric symptoms (hallucinations, paranoia, etc.) they are often unable to participate in decision making in the context of crises (Farrelly et al., 2016). In this context, the Joint Crisis Plan (JCP) has been set up to facilitate shared decision-making and collaboration between patient and clinician (Thornicroft et al., 2013; Bartolomei et al., 2012) in anticipation of potential future crises. JCPs are documents filled out jointly by a person concerned by a severe psychological disorder and/or an addiction problem, a professional and a proxy caregiver if the patient wishes so (Ferrari et al., 2018; Henderson et al., 2015). The tool aims to identify warning signs and triggers of crises and allows the patient to indicate the strategies and resources to be mobilized quickly in the event of a crisis, as well as care and treatment to be favored or avoided (Ferrari & Rouvenaz, 2020). In Switzerland, specifically in the French-speaking part, efforts are being made to disseminate this tool to patients who have experienced psychiatric crises and are likely to experience more of them (Ferrari et al., 2020; Lequin et al., 2021). The JCP-tool helps to avoid rehospitalization and the use of coercion, promotes self-determination, self-care and empowerment, and contributes to psychoeducation (Henderson et al., 2015; Maître et al., 2013).

In French-speaking Switzerland, JCPs are typically paper forms in French, completed during or between consultations and filed in the patient's medical record. Their use is associated with high satisfaction levels among inpatients and outpatients (Bartolomei et al., 2012). However, former research identified issues likely to limit clinicians’ and patients’ engagement in the process and lead to a non-respect of patients’ decisions. Some of these issues are the lack of training in staff carrying out JCPs, a strong asymmetry in knowledge about psychiatry and the JCP, and a fear that patients’ decisions will not be applicable (Henderson et al., 2015; Lequin et al., 2021).

Some authors have explored shared decision-making with patients and proxies who belong to linguistic and cultural minorities and have highlighted difficulties such as the lack of trust in the healthcare system and the language barriers (Hawley & Morris, 2017; Giuliani et al., 2020). However, research on how tools such as the JCP are used with migrant patients remains scarce. We define migrants as persons who live temporarily or permanently in a country in which they were not born and who have acquired important social ties (Bodenmann, et al. 2018). This study aims to investigate clinicians’ and interpreters’ perspectives on the use of joint crisis plans with migrant patients and their proxy caregivers. The objectives are to identify: (1) the linguistic, cultural, and social challenges encountered in the implementation of Joint Crisis Plans (JCPs) with migrant patients; and (2) the practices and resources employed by clinicians and interpreters that facilitate this process.

## Materials and Methods

### Study Design

A qualitative approach enabled a detailed analysis of the issues and strategies relating the elaboration of JCP with migrant patients. The data was produced through collective interviews. Focus groups were ideal for this study, as they facilitate a highly efficient exchange of perspectives on practices and concerns shared by all group members, who stimulate each other in their reflection (Kitzinger, 1995; Krueger & Casey, 2000).

### Data Collection

This study was carried out by a multidisciplinary group of experienced researchers (linguists, nurse, physician, psychologist) within the Community Psychiatry Service of the Lausanne University Hospital. The team also included two external co-researchers: a public service interpreter holding a Federal Diploma of Higher Education who primarily works in psychiatry, and a peer practitioner with lived experience of both mental health challenges and migration.

Four focus groups were conducted with diverse professionals: clinicians using the JCP with and without interpreters; interpreters working regularly in psychiatry with and without experience of the JCP (Table 1). We hypothesized that participants with limited experience, whether with interpreters or with the JCP itself, might express particular concerns regarding aspects of the JCP procedure involving patients with a migration background. Our aim was to be able to document potential differences of this kind.

**Table 1:** Participants of the focus groups

Focus group 1	Clinicians using the JCP with interpreters: 5 nurses, 1 social worker
Focus group 2	Clinicians using the JCP only without interpreters: 2 social workers, 1 physician, 3 nurses
Focus group 3	Interpreters working regularly in psychiatry with experience of the JCP (Portuguese, Kurdish/Arabic, Spanish (2x), Farsi/Dari, Arabic)
Focus group 4	Interpreters working regularly in psychiatry without experience of the JCP (Wolof/Soussou, Spanish, Tamoul, Mongol, Albanian, Tigrinya)

The recruitment of this convenience sample was carried out through the professional network of the research team (colleagues and partners from other institutions). A total of 24 participants were interviewed, 6 in each focus group (see Table 1). They lasted between 1h45 and 2 h, including an introduction that covered two points in order for participants to have a common information base: (1) A definition of “migrants”; (2) An explanation of the JCP-tool and its use in practice.

The discussion was structured around seven questions, developed by OW, VW, PF, and FD to address the objectives of the study. The remaining members of the research team provided feedback on and validated the interview guide. The initial questions explored three stages of the PCC process in relation to migrant patients: participant selection, introduction of the JCP procedure, and the practical development of the JCP. The next three questions focused specifically on linguistic aspects, cultural aspects, and the role of interpreters in the JCP process. The last question targeted ideas for practice. The questions were slightly adapted to the participants’ profession and experience. Follow-up questions addressed insufficiently clear information and fostered examples. The focus groups were led by two members of the research team, one of them leading the discussion, the other taking additional notes to facilitate the analysis. They were conducted between December 2021 and March 2022 and were fully audio recorded and transcribed.

### Data Analysis

The interviews were coded with NVivo, using the principles of content analysis (Mayring, 2000; Neuendorf, 2017). Content analysis inventories and describes the thematic categories emerging from the collected data. In line with an inductive approach, the coding strategy relied on consensus between coders (OW and IS), who collaboratively resolved divergences through agreement on common codes. All members of the research team participated in discussions of the themes and sub-themes that emerged from the coding process.

## Results

The data collected during the focus groups contain seven main themes (see Appendix 1 for more details). The first three themes - Impact of the linguistic situation; Impact of power relations; Cultural representations - refer mostly to difficulties experienced by participants when using JPC with migrant patients and also act as dissuasive factors. The themes 4 and 5 – Translation and adaptation of the JCP tool; Ways of using the JCP with migrant patients – refer to facilitating practices and resources. The two last themes – Involvement of proxy caregivers in the JCP procedure; Roles of the interpreter in the JCP process – deal with issues regarding the involvement of a third party and expose diverse strategies for better collaboration with interpreters.

### Difficulties in the Use of the JCP with Migrant Patients

#### Impact of the Linguistic Situation

According to many research participants, patients’ low French proficiency acts as a significant barrier to communication and strongly impacts not only the procedure but also the therapeutic relationship. Faced with these challenges, some healthcare professionals may choose not to engage in mental health advance care planning altogether (such as JCP) or opt for non-formalized procedures:

“I can do it without it being the JCP. It's more a question of talking about strategies and then discussing it with the patient, but then it's no longer the formal JCP” (FG1^1^)

Clinicians and interpreters’ opinions about the impact of patients’ language proficiencies on JCP discussions differ. Clinicians find it easier to collaborate with patients with intermediate or strong French proficiency than with low French proficiency patients. As long as they approximately understand each other, they prefer working without interpreters:

“Not speaking French adds the burden of having to deal with an interpreter because sometimes it takes time to explain the process, why we ask these questions, […] well I’m a bit hesitant to offer that to these patients” (FG2).

On the other hand, interpreters find it easier to complete a JCP-form with low French proficiency patients who systematically have interpreter mediated consultations. With patients with intermediary French, interpreters are often called only once the clinician realizes there are communication issues with the patient, which represents an additional challenge:

“And then, […] they called me after they already started to fill in the document [without an interpreter]. […] I had to stick to a questionnaire without really knowing what had happened before. […] So for me it's a bit like I jump out from nowhere [...] It's confusing not only for me, but also for the patient” (FG3).

The majority of participants mentioned the necessity to work with an interpreter as often as possible with low and intermediate French proficient patients, in order to help them express their emotions and ideas in detail. Dealing with non-professionals (eg. a relative) should be avoided because of their emotional involvement and their limited interpreting skills. However, collaborating with professional interpreters alone does not solve all communication issues. Some interpreters judge the medical discourse of the JCP too complicated for the patients even if it is translated into their language. This might in part explain why, on the other hand, several clinicians feel that original speech from both sides is impoverished by the process of language interpreting. In some cases, the time added to the discussion due to the process of interpreting seems to negatively affect the quality of JCP discussion because clinicians tend to hurry in their work in order to make up for the time that is “lost”.

#### Impact of Power Relations

Institutions – not only in the health sector – tend to discriminate minority populations. This goes along with specific power issues in cross cultural mental health care. Indeed, migrants may have experienced abuse of power and discrimination in their host and their home countries, which impacts their relationship with professionals. An accumulation of coercive experiences in various environments leads to massive mistrust, but also to a strong fear of negative consequences of not collaborating with institutional representatives. For this reason, participants frequently felt that migrant patients were reluctant to the JCP-form, but still gave their consent.

“Sometimes patients say yes but it doesn’t really make sense to them” (FG1)

As reported by several participants, the act of writing personal matters about mental health on a document and signing it can be very stressful for patients who conceive the health sector and other administrative authorities as closely interconnected.

“It's a document that you sign and for most of our patients who have a link with institutions, there's a confusion between, I don't know, the state and the care services. Putting things on paper is sometimes experienced as a threat or as if they were signing a contract“. (FG3)

Patients seem namely to fear undesirable interference between JCP and administrative procedures for residence or refugee permits:

“[…] when there are stories of persecution in the past […] if you write down [mental health problems], they say that it will pursue them: “what happens with that? I’ll be expelled” […]. And it’s not always easy for them, well, they can be ok in principle, but when they are faced with the thing […] it stresses them out”. (FG1)

In addition, precarious living conditions of migrant patients may complicate the JCP procedure. Clinicians report that part of their migrant patients lack the resources required for a minimum of socio-economic stability. As a result, some clinicians feel that JCPs are not a priority and do not engage in this process. The socioeconomic precariousness may therefore deprive impoverished patients facing deportation risks of access to procedures intended to foster their empowerment within the clinical setting.

#### Cultural Representations

Some participants mentioned that cultural differences between health professionals and some groups of migrants complicate the use of JCP. The cultural background influences explanatory models of illness and ideas about effective care. It also shapes the very expression of mental illness:

“In the community for which I translate, the notions of thoughts, emotions, physical reactions, behaviors always lead to confusion. I often meet people for whom the notion of emotions is not as clear as it seems for the Western communities. […] The people I meet most often talk about physical reactions” (FG4)

According to several clinicians and interpreters, religious and traditional beliefs are central to the way suffering is explained (e.g. black magic). Reluctance to complete a JCP may also be tainted by the belief that talking about the crisis could trigger future ones. Some patients appear to have a negative opinion on psychiatry which they consider to be “for crazy people”. This judgement can cause resistance towards care.

With regards to treatment, representations of the roles and responsibilities of the professional and the patient often complicate the process. Some participants mentioned a paternalistic view of medicine among some groups of migrants, making pointless a process of shared decision-making like the JCP.

"The first generations of Albanians from Kosovo are used to a rather paternalistic medicine. And so, if they were confronted to a JCP, the comment they would automatically make […] would be "But what am I here for? Are you not the one who's going to answer all these questions?" (FG4)

On the other hand, it was pointed out that professionals and institutions have their own, Western, models of understanding mental illness and roles, and that tools such as the JCP reflect this professional culture. In practice, clinicians consider that migrant patients are particularly in need of knowledge about the health care network, and they suggest that a more common view between clinicians and patients should be reached by the provision of specific explanations:

“I noticed something, and this is also true for the Swiss, but it's even more true for the others, it's the lack of knowledge of the network [...] So there's an extra layer of explanation of how it works, where to go for what” (FG1)

### Facilitating Practices and Resources

#### Translation and Adaptation of the JCP Tool

The lack of medical documents (e.g. JCP) translated into migrant patients' first languages is repeatedly cited as an institutional barrier to quality care. Translating the JCP form in foreign languages would not only favor its use for clinicians and patients but would allow interpreters to prepare their interventions.

“What I found difficult was having to translate all the questions that had already been thought of a lot. [...] So if these documents could be translated for us in advance, to prepare [...], then it's much easier to [...] present this JCP, the objective, and then continue with the questions”. (FG3)

Some participants suggested the development of a bilingual document, in which all questions and answers are written in French and in the patient's language, in order to be understandable for all the users of the JCP.

Several interpreters and clinicians claimed that in addition to translation, the text of the JCP form needs cultural adaptation. Some complex medical concepts should be simplified. Above all, interpreters stressed the importance of including examples of therapeutic options and other aspects that may be too vague for some patients.

“The migrants don't know what kind of offers exist here. Is it medication? Is it psychological support? What kind of care is the person entitled to? […] So maybe simply adding examples so that the [patient] can choose and can say to themselves "oh yes, we're talking about this, okay I understand, this I don't want, this I want”, etc". (FG3)

Finally, a task force should create an explanatory document that could promote the JCP as a formal and standardized tool and not a document the clinicians improvised themselves in order to control the patient.

“I think that it should be translated in several languages with perhaps an explanatory document, also translated, so that the patients feel that it is something consistent and that it doesn’t depend on the clinicians [...] “he did not impose this on me [...] he’s using it but did not invent it”” (FG2)

### Ways of Using the JCP with Migrant Patients

To facilitate the use of JCPs with migrant patients, participants identified strategies for clinicians that lead to a better relationship with the patients. Most importantly, before introducing the JCP to the patient (introduction phase), professionals should pay close attention to timing issues. Indeed, it is essential to reach a trusting relationship with the patient, and make sure their situation is sufficiently stable. In some cases, patients need to see that crises are recurrent in order to understand why a crisis plan is necessary. When patients face acute social issues (e.g. threat of expulsion; housing problems), clinicians should pay particular attention to the sense of the JCP process for the patients. If these timing issues are neglected, the JCP loses its efficiency.

“It's true that I usually take a little time to create a relationship and get to know them to see where their needs are, their requests, to try to meet them. Finally, it also allows me to highlight whether it makes sense to do it or not in their current situation. But it does take time to get to know each other”. (FG1)

During the elaboration of the JCP (elaboration phase), participants suggest that clinicians should take time to explain concepts that patients might not know (e.g. distinction between emotions, thoughts, behaviors, and physical reactions) and adapt their explanations to each patient. This implies giving examples of possible answers to the JCP questions, namely by presenting different options of dealing with psychiatric crises. In practice, this requires longer consultations and gives the patient more time to reach a positive decision on JCP participation. This whole process should be conducted with an interpreter when common language skills are too scarce. All these requirements impose major flexibility on the clinicians, who have to develop an individualized approach for patients.

There were differences of opinion about who should write the JCP. Some participants expressed a preference for the patient, since they felt that this promotes their empowerment; others suggested the clinicians, in order to provide the patient with more freedom to discuss; and other participants mentioned the interpreters, with regards of their language skills.

When the JCP is completed and ready to apply in case of crises (application phase), a copy of the document should be given to the patient. This should allow its application even in case of a remigration:

“But there are migrants who come here and then tomorrow they will be in Germany. And then they decompensate and end up here, and we try to follow them up, and then they disappear” (FG2)

### Third Parties in the JCP Process with Migrants

#### Involvement of Proxy Caregivers in the JCP Procedure

During the JCP procedure, clinicians are expected to investigate if patients wish to associate members of their personal network as partners in the crisis plan. According to the focus groups, migrants are more likely to experience isolation and to be deprived of a social network. Moreover, participants added that some patients prefer to hide their mental health issues or exclude their relatives from the procedure. This may be due to the desire to hide a failure of the migration project, but also to the fear of being stigmatized. For the second reason, clinicians may also be reluctant to work with potentially counterproductive families.

“I find that the complexity of including the family is that they themselves may have this [negative] representation [of psychiatry]. I would see the risk that [the JCP] may be used by the family as a tool to constrain the user” (FG2)

Some participants said that it is therefore necessary to work with the patient on this feeling of shame and on the possibility of asking proxies for help.

#### Roles of the Interpreter in the JCP Process

The focus groups discussions suggest that interpreters endorse a multiplicity of roles in JCP procedures. Interpreters describe themselves primarily as someone who translates, but is also able, on health professionals’ request, to act as a cultural informant, e.g. by explaining the dominant representation of psychiatry in the patient's community. In addition, interpreters simplify or rephrase health professionals’ and patients’ words so that they can understand each other. In this case, they often add examples. Several interpreters found it difficult to translate as accurately as possible, while adapting speech to the cultural background of its recipient. Some of them would therefore prefer that health professionals take the responsibility to simplify their words for the patients.

“I don't feel capable of translating this to someone. And actually it's not up to me as an interpreter, it's up to the doctor to explain this document, what it means.” (FG4)

Another role taken on by interpreters is that of facilitator of the patient's trust in the health professional. The presence of the same interpreter throughout the whole procedure represents a prerequisite for this role:

“By being a little more than an interpreter, you can have their confidence. […] it's true that in the balance you have to be really neutral and impartial, but you have to be a bit on the migrants' side.” (FG3)

It appears that among our participants, only interpreters mentioned this role of facilitating patients’ trust. Clinicians did not seem to be aware of it.

Several members of the clinician focus groups consider interpreters contributions as cultural informants as helpful. However, some of them find it disturbing when interpreters leave their role as language translators without discussing it with the clinicians, especially when they develop a too close relationship with patients. On the other hand, interpreters point at clinician behavior making it difficult to stick to a translator role and undermining patients’ trust:

"When we're talking to the doctor, who tells us what's going on for a long time, sometimes they don't respect the fact that we ask them not to speak in too long sentences. So there you have it, the immigrant is there, he listens, he understands absolutely nothing of what's going on and then sometimes, I had the impression that [the patients] were suspicious" (FG3)

For all these reasons, many participants find meta-communication between the clinicians and interpreters necessary. Interpreters insist on having time to coordinate with the health professionals and to prepare for the translation of some contents. Some participants pointed out that training interpreters in the use of JCP tool would facilitate their collaboration with health professionals. The need for post-consultation debriefing was mentioned in order to report difficulties or misunderstandings. Finally, an interpreter suggested that health professional-interpreter collaboration should last until or even after the application phase of the JCP, so that the interpreter is not left alone with the patient in the event of a crisis. This implies that at least some of the interpreters view themselves as possible first interlocutors for patients trying to apply the crisis plan.

“This makes me think of the question of resource persons, well they often name the interpreter. And the interpreter's role is to interpret and not necessarily to be there for the person, and there are many who find themselves in situations that are not easy in fact.” (FG1)

## Discussion

The results revealed that the use of JCPs with migrant patients is complex, because of linguistic, cultural and power aspects. Barriers in these areas affect all phases of the JCP procedure (selection, elaboration, application). This suggests that the JCP as an advance care planning tool is not easy to adapt to migrant populations. As a result, clinicians are often discouraged and tend to exclude migrants from JCPs.

The results showed that the absence of a common language between clinicians and patients impacts the JCP procedure and the therapeutic relationship. Therefore, working with interpreters appears to be a necessity to improve the quality of clinical care for non-native patients with low proficiency in the local language (Karliner et al., 2007). However, despite clear evidence of their positive contribution, interpreters remain underused in clinical contexts (Diamond et al., 2009). According to Roberts (2022) and Wurth et al. (2018), the decision whether to use an interpreter is quite complex “because everyone is in a continuum of linguistic competence, so the conditions for negotiating shared understanding can be elusive” (Roberts, 2022). In practice, clinicians tend to ignore conversational limits and only use an interpreter for “high stakes” conversations (Brandenberger et al., 2019).

Participants also discussed about the migrants experiencing power abuse and coercion in mental health care. These negative experiences cause mistrust against health professionals (Silva et al., 2023). Some migrant patients may give their consent to the JCP only because they fear consequences on their administrative procedures. According to Hynie (2018), groups of people that are victim of power abuse and discriminations are more likely to develop mental health issues. Asylum seekers are particularly vulnerable as they await the review of their claim and at the same time face restrictions on access to employment, housing, and other conditions of residence (Hynie, 2018). Patients in administrative proceedings are not willing to respond to mental health care offers because they focus primarily on socio-economic stability (Satinsky et al., 2019). It results in inequality in terms of access and quality of care for migrant patients, some of whom seem hard to reach.

Finally, some findings highlight the influence of migrant patients' cultural backgrounds on their explanatory models, idioms of distress, and the effectiveness of psychological interventions within culturally diverse groups. There is a vast body of literature on the difference in symptom expression across cultures, e.g. with regard to emotional vs somatic complaints (Kirmayer, 2001; Ma-Kellams, 2014). That is why some authors work on culturally adapted interventions such as the cultural formulation interview, to investigate patients’ understanding of their problem and focus on their social context (Heim & Kohrt, 2019; Jarvis et al., 2020). Results also suggest that cultural representations influence expectations regarding the style of consultations. Indeed, shared decision-making models may not resonate with patients whose experience with health professionals does not include sharing information or discussing their treatment preferences (Roberts, 2022). It is the case with some groups of patients who expect health professionals to decide for them. Cultural barriers thus influence the ability and desire of patients to engage in JCP procedure. As a result, they seem disadvantaged when it comes to advocating for their healthcare and this reinforces inequalities of care for the migrant population (Hawley & Morris, 2017; Fennig & Denov, 2021).

However, it is worth noting that the difficulties in the JCP procedure with migrant patients are not only related to linguistic and cultural aspects. Farrelly et al. (2016) have identified limitations regarding the JCP procedure with mainstream patients. They mentioned that JCP-tools are often designed to meet the needs of the mental health services more than the needs of patients. Also, patients and clinicians happen to have different perceptions regarding the JCP: while clinicians feel they are engaged in a shared decision-making process, patients feel excluded from decisions (Ferrari et al., 2018). Therefore, significant power differences between clinicians and patients complicate the shared decision-making approach in mental health. Moreover, patients – both migrant and non-migrant – often exclude their proxies from the procedure because they fear disclosing personal information about their mental health issues (Lequin et al., 2021; Ferrari et al., 2018).

In line with the literature on interpreting in other mental health settings (Brisset et al., 2013; Fennig & Denov, 2021; Hsieh, 2010), participants repeatedly mentioned professional interpreters as having a central role in overcoming the barriers mentioned above. However, it seems that the interpreters' skills are not sufficiently exploited, especially because of an unsuccessful clinician-interpreter cooperation, and also limiting settings (lack of time, complex language in documents, etc.). Brisset et al. (2013) mentioned that collaboration difficulties with interpreters are often caused by clinicians’ fear to lose control and authority over the patients. Fiona Wood et al. (2017) mentioned the complexity of introducing a written document in a translated consultation, without previous instructions and negotiations. It all results in an incomplete procedure, dissatisfaction and divestment of actors. This study demonstrated interpreters acting as facilitators of a trusting relationship. Leanza et al. (2015) showed that the presence and activities of interpreters in mental health interventions have some therapeutic value, because of their ability to create a working alliance based on trust, respect and recognition. However this role is only possible within a defined framework and can be prevented by clinicians’ fear of losing control, lack of time and continuity or role ambiguity. The non-integration of interpreters into a common defined framework may increase transgression of limits for interpreters who take on roles that are not part of what is expected of them.

Among the strategies explored to facilitate JCP access for migrant patients, translation and cultural adaptation of the document appeared to be a necessity. In regards to advance directives, most health care services require written documents for legal matters. A translation of the document should ensure their validity and clinical usefulness (Castillo et al., 2011).

However, the results of this study left some questions unanswered regarding the actual drafting of a JCP in multilingual consultations. What does not seem to be clear to the participants is who writes the JCP first (clinician? interpreter? patient?), whether there should be one or two versions ("first language" *and*/*or* "French") and how to ensure that the patient has a clear understanding of the final document. Future work should focus on developing practical guidelines for writing JCPs and translation rules in multilingual context. However, research-based formal guidelines for writing the JCP are also lacking in monolingual contexts (Ferrari et al., 2018).

### Implications for Practice

Due to the limited data of this study, preliminary recommendations for practice have been developed. To facilitate access to JCP and more globally to mental health care for migrant patients, several actions at both institutional and clinical levels need to be considered. At the institutional level, translation of documents in several languages must be carried out. Guidelines should be developed on the writing of medical documents (e.g. JCP or other advanced measures documents) with patients consulting with interpreters. They should contain information on the translation and drafting process, as well as on the monitoring process during the application phase (Farrelly et al., 2016). Training for interpreters on the use of tools of advance care planning or advance directives should be considered. At the clinical level, it is necessary for the clinician to adapt the procedure according to the patient’s language proficiency, cultural background and health literacy. The clinicians should consider with great care the patient’s representations and expectations and take time to discuss about them. They should have the opportunity to work on the patient’s negative emotions (e.g. fear, mistrust, shame) that cause resistance. Health professionals must be aware of their own cultural background, both personal and professional, which allow to take a step back from one's own practice and from what can block the relationship with the patient. Linguistic needs are central in patient’s care and must be taken seriously by using professional interpreters as often as possible (even with patients with intermediary French proficiency) and even before introducing the JCP to the patient. It is therefore necessary to improve collaboration between clinicians and interpreters by doing briefings, to agree on each other’s roles (Delizée & Michaux, 2022; Weber et al., 2023).

In conclusion, the use of JCPs in multilingual and multicultural settings requires a high degree of flexibility from clinicians. This can be challenging, given that the tool is designed to be standardized and applied uniformly across patients. Nonetheless, such adaptability is essential for delivering high-quality, person-centred mental health care.

### Limitations

First of all, despite the definition of migrants exposed to the participants during the focus groups, the clinicians referred most of the time to patients from a distant country and living in precarious conditions. Migrants from Europe who have been living in Switzerland for a long time have rarely been considered and the use of JCPs with those patients would probably deserve more attention. Second, the data collected in this research are limited to the experience and opinion of a limited number of clinicians and interpreters. Due to the recruitment of participants mainly within the authors’ networks, some problematic aspects of the use of JCP may have been mitigated or overstated during the focus groups for reasons of social desirability.

Further research should be conducted with larger and more independent samples, less closely associated with the authors. It should also focus on migrants’ experience in JCP from their own viewpoints and, more generally, their representations of clinicians and health care. Finally, this research does not draw on direct observation of practices regarding JCP with migrant patients. Ethnographic research on JCPs and other forms of advance care planning in mental health with migrant patients should complement this study.

## Conclusion

This study provides original insights into the use of joint crisis plans with migrant patients. Social precariousness, availability of linguistic resources –oral and written–, and cultural representations of illness and health feature complicate JCPs with some groups of migrant patients. Several strategies would help clinicians adjust their practice according to the different actors involved. They are costly in terms of time and effort but facilitate more solid and trustful –dyadic or triadic– relationships with patients using JCPs.

## Appendix 1

**Table:**

**1) Impact of the linguistic situation**
Language situation may block JCP procedure
Complexity when patient and clinician understand each other
Problems when non-professionals translate
Added value of the interpreter
Problems because of or despite a professional translation
**2) Impact of power relations**
Abuse of power and discrimination in the health care system
Migrants’ precarious living conditions of migrants
**3) Impact of cultural representations**
Patients' religious and traditional beliefs
Cultural idioms of expression of suffering
Negative representation of psychiatry
Cultural representations of illness and health
Lack of knowledge of the health care system more present among migrants
Challenges related to roles and responsibilities (divergent views)
Preference for male or female carers
Cultural anchoring of the PCC
**4) Translation and adaptation of the JCP tool**
Change the terms to make JCP less “scary”
Medical language too difficult to understand
Document too short to integrate the content in both languages
Create a translated explanatory document to formalize the tool and avoid the feeling of constraint
Include examples of available offers in the PCC to facilitate translation
Need to translate the JCP beforehand to prepare the interpreter
JCP adapted to the patient's culture
JCP translated into several languages
Difficulty in standardizing a document while adapting to the patient
**5) Ways of using the JCP with migrant patients**
Preparation for the JCP
Practice during the completion of the JCP
Transition from oral to written
Validation and implementation of the JCP
Patient education
Integrating patients' cultural representations
**6) Involvement of proxy caregivers in the JCP procedure**
Families rejected to hide the failure of the migration project
Families too counterproductive to be involved in the JCP
Notion of sharing with relatives in the JCP as a reason for migrant patients to refuse
Refusal to share with the family some of the topics discussed
Working with the patient on the possibility of asking for help
**7) Roles of the interpreter in the JCP process**
Cultural informant
Interpreter fills in JCP with patient without clinician
Interpreter as writer when patient cannot write
Interpreter's relational role
Translate accurately the meaning of what is said
Vulgarisation and cultural adaptation of what is said

*JCP* Joint Crisis Plan

## Footnotes

1 FG = focus group.

## Declarations

### Research Involving Humans

This study was performed in accordance with the ethical standards of the Commission for Ethics in Research on human beings of Vaud (CER-VD), within the framework of request no. 2016–00768 “Systemic evaluation of the community shift in French-speaking Switzerland”.
